# Mirizzi Syndrome: A Case Report and Review of the Literature

**DOI:** 10.7759/cureus.24375

**Published:** 2022-04-22

**Authors:** Imran Khokhar, Maija Adourian, Eldia Delia, Gisha Mohan, Mathew Mathew

**Affiliations:** 1 Internal Medicine, Suburban Community Hospital, Norristown, USA

**Keywords:** chronic cholecystitis, jaundice, csendes classification, gallstones, cbd, mirizzi syndrome

## Abstract

Mirizzi syndrome (MS) is a rare complication of chronic gallstones. Mirizzi syndrome is characterized by a set of symptoms that results from obstruction of the common hepatic or common bile duct (CBD). This may be due to extrinsic compression from an impacted gallstone in the gallbladder neck or cystic duct because of inflammatory changes secondary to chronic gallstone cholecystitis. We present a case of an 86-year-old patient with chronic gallstones who presented with abdominal pain and jaundice. The patient was diagnosed with MS type V after endoscopic retrograde cholangiopancreatography (ERCP). CBD stone fragments/debris were removed, and the patient was referred for surgical intervention for the repair of cholecystoduodenal fistula. MS must be in the differential diagnosis in elderly patients with chronic gallstone cholecystitis presenting with obstructive jaundice. Multiple diagnostic and therapeutic approaches are required to diagnose and manage the different types of MS. We aim to present the case to highlight and raise awareness of MS, particularly in patients with chronic gallstones.

## Introduction

In 1948, Mirizzi syndrome (MS) was first described as a rare complication of long-standing gallbladder stones. It refers to the set of symptoms that result from obstruction and fistula formation of the common hepatic or common bile duct (CBD) due to long-standing gallstones. This may be due to extrinsic compression from an impacted gallstone in the gallbladder neck or cystic duct because of inflammatory changes secondary to chronic gallstone cholecystitis. Over time, the stones can erode and form cholecystoduodenal or cholecystoenteric fistulas which further complicate the pathology. In patients presenting with obstructive jaundice or ascending cholangitis, it is crucial to have a high index of suspicion for MS, as it could be easily missed [1]. MS must be in the differential diagnosis in elderly patients presenting with obstructive jaundice, in addition to gallbladder cancer, cholangiocarcinoma, pancreatic cancer, sclerosing cholangitis, and metastatic disease [2]. Distinguishing between these conditions can be challenging, but accurate diagnosis and appropriate management result in significantly more favorable outcomes [3].

## Case presentation

An 86-year-old male with a past medical history of recurrent gallstone cholecystitis presented in the emergency department (ED) with intermittent right upper quadrant abdominal pain, diarrhea for the last two to three weeks, and scleral icterus. His medical history was remarkable for chronic lower extremity edema, deep venous thrombosis with inferior vena cava filter, and incision and drainage of septic arthritis of the right hip. The patient was unable to provide detailed history secondary to poor memory; history was ascertained from family and electronic medical records. 

One year ago, the patient had similar abdominal pain and a computed tomography (CT) scan of the abdomen and pelvis showed a distended gallbladder with cholelithiasis, including one stone possibly impacted in the gallbladder neck. He subsequently underwent a hepatobiliary iminodiacetic acid (HIDA) scan which confirmed an obstructed cystic duct stone. The patient was medically not stable for the surgical approach and interventional radiology placed a percutaneous cholecystostomy tube. The patient received intravenous antibiotics and was clinically improved. One month later, the patient underwent a cholangiogram which revealed a patent biliary duct, confirming the safe removal of the cholecystostomy tube. He was advised to undergo elective cholecystectomy at that time, which he declined and did not follow up. 

During this admission, the patient complained of intermittent severe abdominal pain. There were no aggravating or alleviating factors associated with the pain. He denied weight loss, nausea, emesis, chest pain, and shortness of breath. In the ED, vital signs were remarkable for hypotension and tachycardia. On physical examination, the patient was alert and oriented to self. Pertinent physical findings include scleral icterus and tenderness in the right upper abdomen.

**Figure 1:**
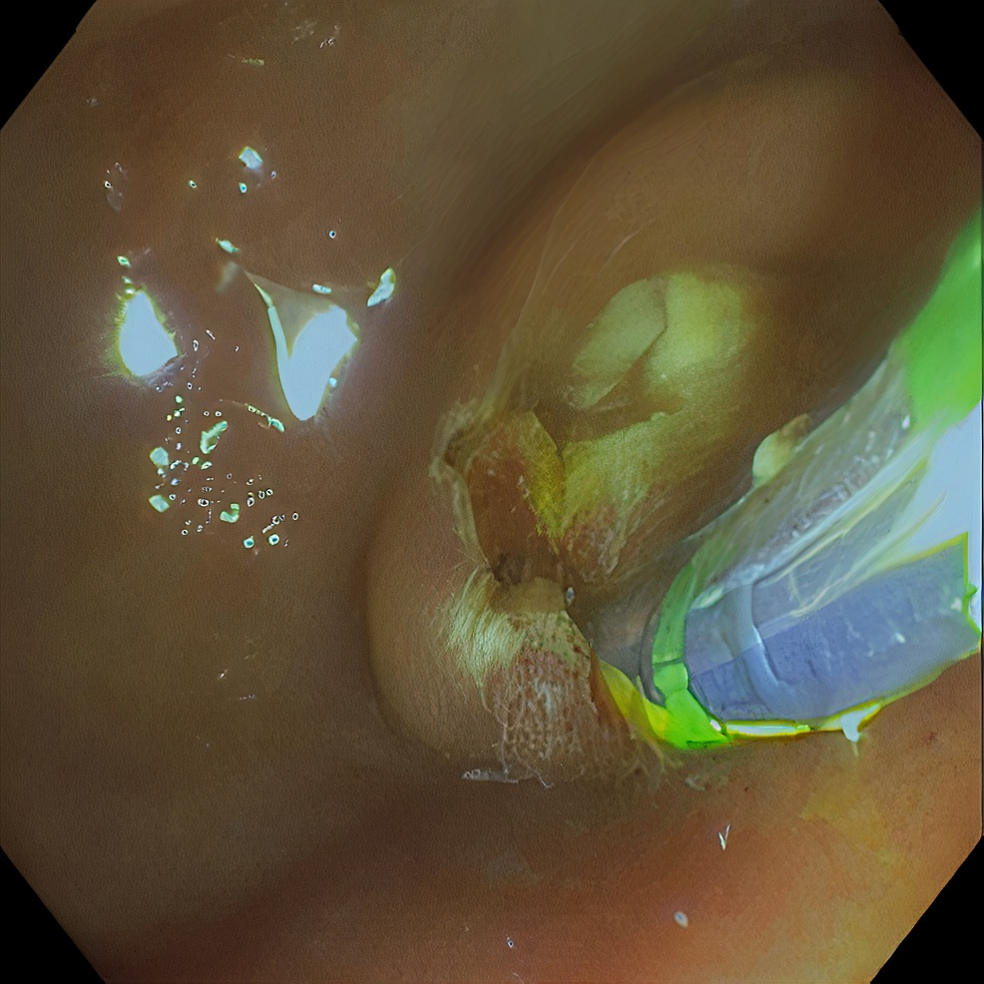
ERCP guided sphincterotomy. ERCP: Endoscopic retrograde cholangiopancreatography

**Figure 2:**
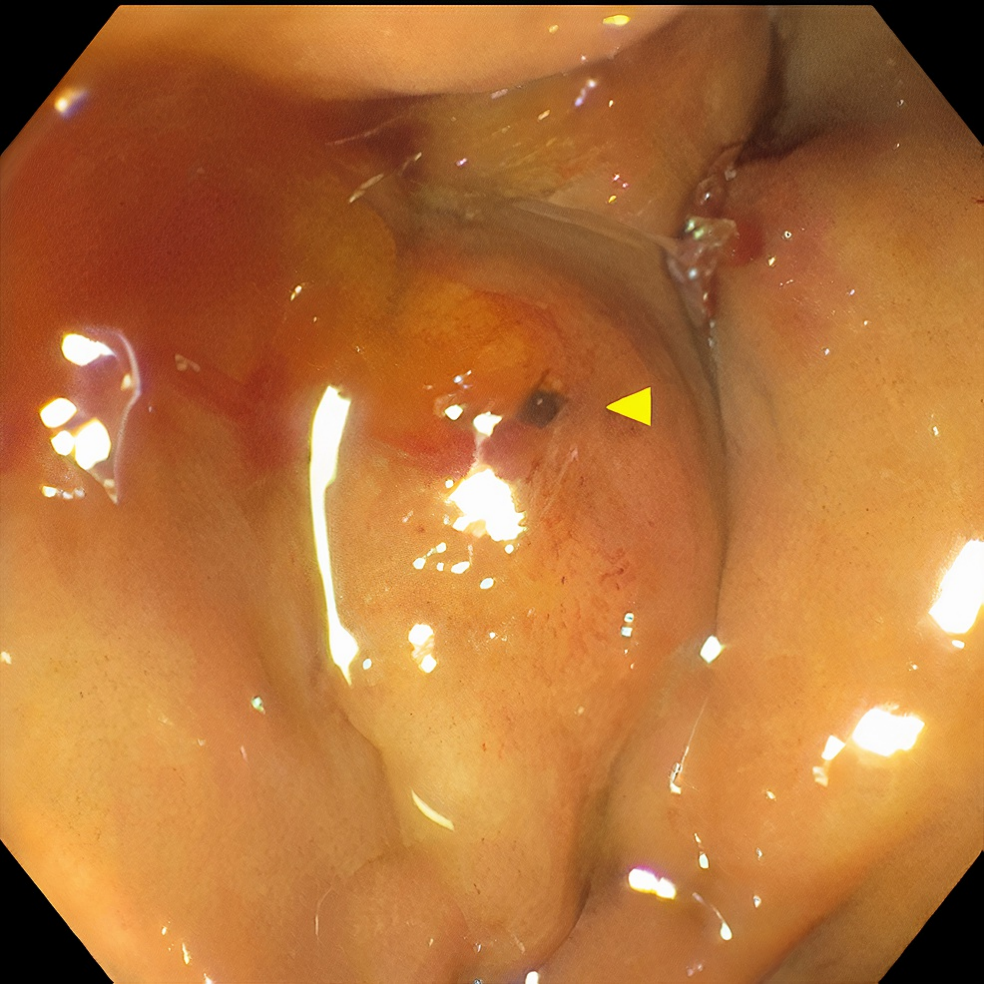
ERCP showing fistula between gallbladder and duodenum. ERCP: Endoscopic retrograde cholangiopancreatography

**Figure 3:**
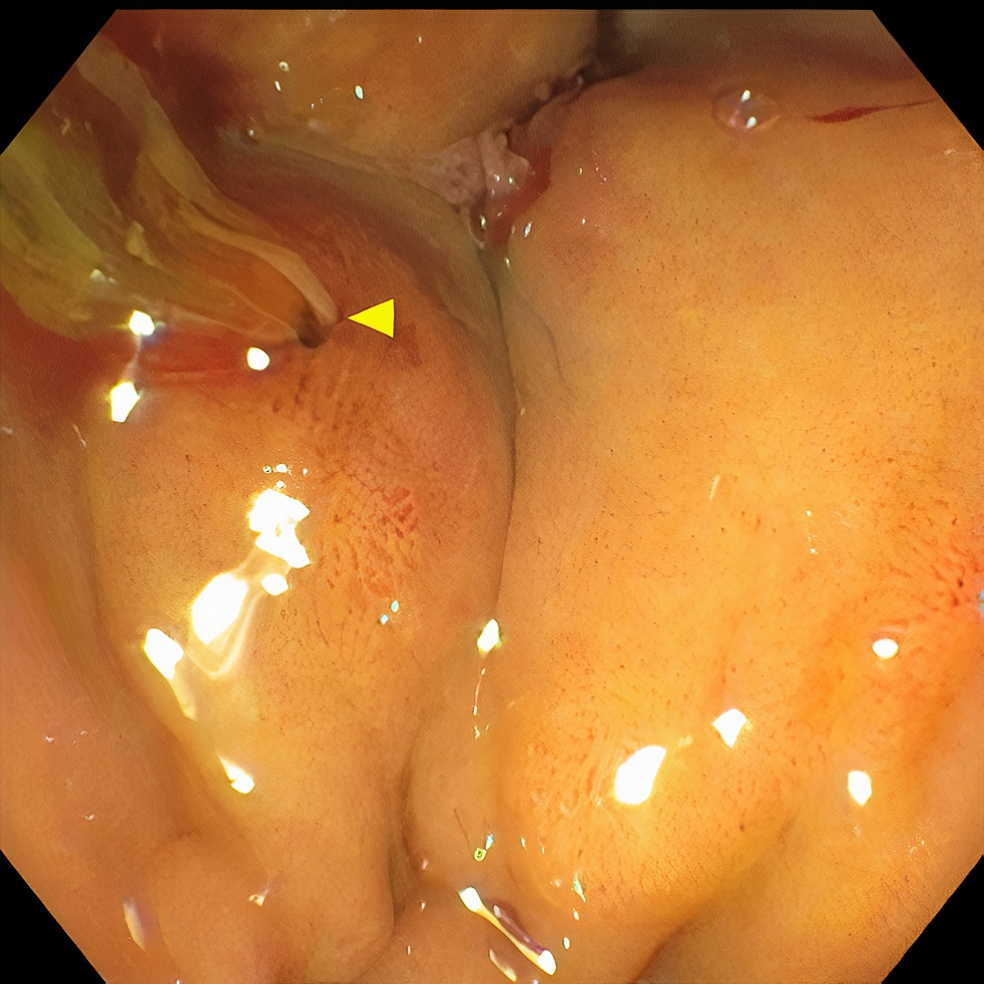
Drainage of pus from fistula between gallbladder and duodenum during ERCP. ERCP: Endoscopic retrograde cholangiopancreatography

**Figure 4:**
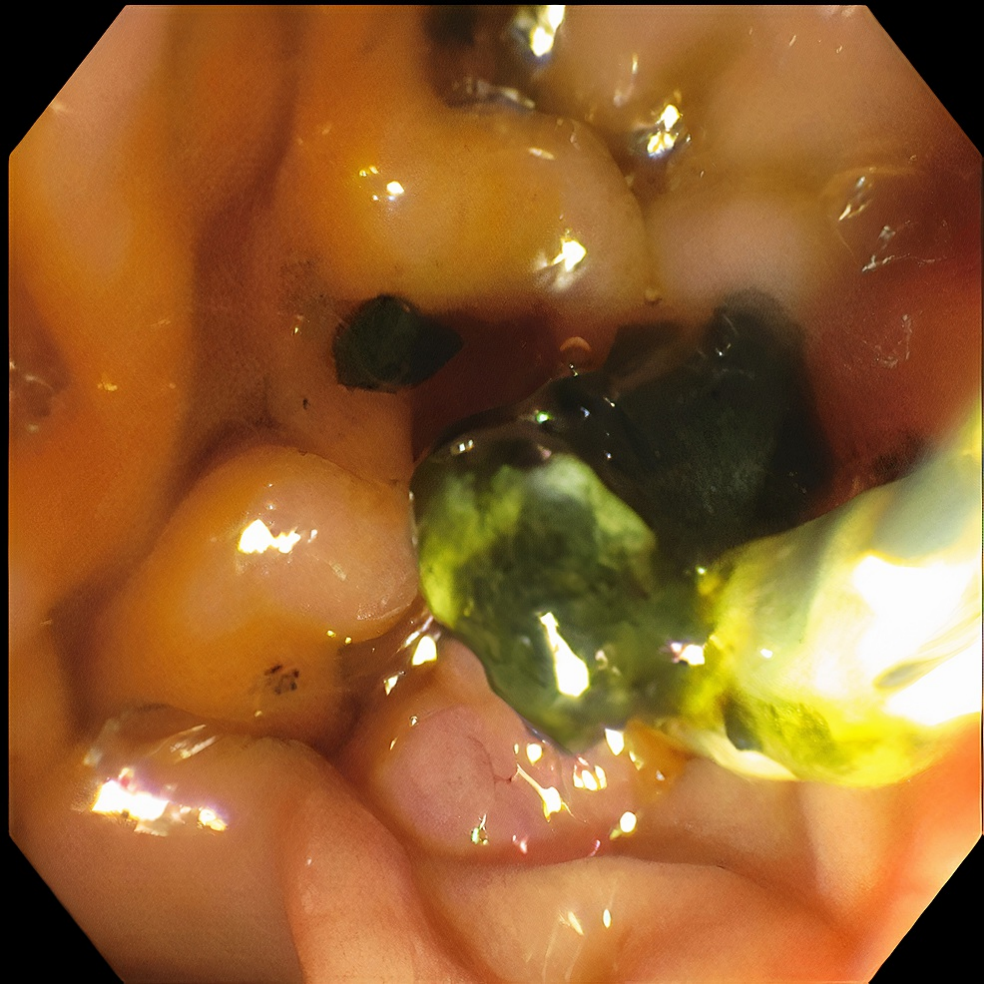
Removal of stone debris during ERCP ERCP: Endoscopic retrograde cholangiopancreatography

Pertinent labs include elevated white blood cells (13.5x10³/µL), total bilirubin (15.7 mg/dL), direct bilirubin (11.7 mg/dL), aspartate aminotransferase (98 U/L), and alanine aminotransferase (41 U/L). Urinalysis showed tea-colored urine. CT scan of the abdomen and pelvis revealed a collapsed gallbladder with CBD obstruction and bile duct dilation consistent with chronic inflammation. Endoscopic retrograde cholangiopancreatography (ERCP) was then performed which demonstrated cystic duct stone eroding into the common hepatic duct, hence the diagnosis of Mirizzi syndrome was made. Cholangioscopy confirmed the eroding stone, chronic inflammatory changes, and destruction of the bile duct. A cholecystoduodenal fistula with pus drainage was noted in the duodenal bulb. The patient was diagnosed with MS type V according to the ERCP findings. The stone was not amenable to endoscopic removal, but stone debris, clots, and pus (Figures 1-4) were swept. He was referred to the surgical team for cholecystectomy and repair of cholecystoduodenal fistula.

## Discussion

The prevalence of gallstones in the United States is roughly 15% [4]. Factors that increase the risk for gallstones include female gender, age >40 years, obesity, high-fat diet, family history of gallstones, diabetes, and estrogen-containing medications. Most gallstones are asymptomatic, referred to as silent gallstones. About one in five will develop symptoms, and presentation varies depending on the number, size, and location of gallstones [5]. The most common presentation includes sudden, intense pain in the right upper quadrant radiating to the right shoulder. Associated symptoms include nausea, vomiting, fever with chills, and scleral icterus. Acute cholecystitis/biliary colic may follow fatty meals and mostly occur in the evening or during the night [6,7]. Most of the time, biliary colic resolves when the gallstones move and no longer block the bile ducts. Once gallstones become symptomatic, there is a greater chance of recurrence. If gallstones become symptomatic and there are recurrent episodes, more definitive treatment may be warranted. There are different procedural options including endoscopic retrograde cholangiopancreatography (ERCP), shock wave lithotripsy, laparoscopic cholecystectomy, open cholecystectomy, and robot-assisted techniques [3]. Prevention of gallstones requires a diet high in fiber but low in fats, refined carbohydrates, and sugar. Slow weight reduction is important in obesity, as rapid weight loss can exacerbate gallstone formation. In addition to maintaining a healthy weight with lifestyle and diet modifications, sometimes medical therapy is used for a single symptomatic gallstone [4].

Complications of gallstones are numerous and can involve the gallbladder, biliary tract, and intestinal tract. Symptomatic gallstones can cause acute or chronic cholecystitis, empyema, perforation, pericholecystic abscess formation, adenocarcinoma, ascending cholangitis, obstructive jaundice, Mirizzi syndrome, gallstone ileus, and Bouveret syndrome [8]. Over time Inflammation can cause cutaneous fistulas, secondary biliary cirrhosis, biliary strictures, and even death [3]. Mirizzi syndrome is the most uncommon complication, which is difficult to diagnose and treat, making it challenging for a biliary surgeon. Epidemiological studies in patients with cholelithiasis show that only 0.1% developed MS. Prevalence of 0.7%-25% is noted in patients who have undergone cholecystectomies [7]. The Csendes classification system of MS is the most widely accepted [9].

**Table 1:** Csendes classification system of Mirizzi syndrome References [9-11]

Type	Description	Incidence (%)
I	Stone in the cystic duct or neck of gallbladder extrinsically compresses common hepatic duct or common bile duct	10.5-78
II	Cholecystobiliary fistula involving less than ⅓ of the circumference of the common bile duct	15-41
III	Cholecystobiliary fistula involving between ⅓ and ⅔ of the circumference of the common bile duct	3-44
IV	Cholecystobiliary fistula involving the entire wall of the common bile duct; complete fusion causing no recognizable dissection planes between both biliary tree structures	1-4
V	Cholecystoenteric fistula plus any other type of MS	29

For patients with MS, endoscopic intervention and surgery are the mainstays of treatment for patients who are surgical candidates [3,12]. In terms of which surgical approach is most appropriate, many factors must be taken into consideration. The patient’s age, MS type, and cardiac/respiratory comorbidities all play a role. However, ERCP with stenting and endoscopic lithotripsy are sometimes attempted prior to other surgical approaches [3,12,13]. Surgery on these patients is complex for various reasons: 1. Preoperative diagnosis is often missed due to the low index of suspicion and rarity of this condition [1,3,12]; 2. Distorted anatomy due to the dense adhesions that have built up over time from chronic cholecystitis [3]; 3. Cholecystobiliary or cholecystoenteric fistulas (MS), which increases the risk of bile duct injury or hemorrhage while dissecting the Calot triangle [3]; 4. Impacted cystic duct stones tend to be inaccessible or irremovable endoscopically [12].

Hepatobiliary surgeons must carefully assess the clinical situation and anatomy when dealing with MS. The most common and expected surgical complication includes direct intraoperative biliary injury. A surgical complication can be minimized by the preoperative placement of a stent via ERCP [5]. Different treatment options depending upon the classification/type of MS are demonstrated in Table 2. 

**Table 2:** Treatment options for each type of Mirizzi syndrome. References [1,3,12]

Type	Treatment Options
I	Total cholecystectomy (open or laparoscopic)
II	Partial cholecystectomy or fundus first technique
III	Partial cholecystectomy or fundus first technique
IV	Cholecystectomy and Roux-en-Y hepaticojejunostomy (RYHJ)
V	Laparotomy with cholecystectomy/RYHJ and repair of fistula

Overall MS is challenging to diagnose and treat. The diagnostic difficulty is frequently experienced as MS can mimic cancer of the gallbladder. Patients with MS also have an increased risk (5%-28%) of developing gallbladder cancer, likely from chronic biliary stasis causing persistent and recurrent irritation of the area [7]. Although CA 19-9 can be used to differentiate between MS and gallbladder cancer, it is not definitive. There have been MS cases reported with high levels of CA 19-9 in the absence of malignancy [14].

In patients with MS, preoperative CT, and magnetic resonance cholangiopancreatography (MRCP) should be done to rule out gallbladder cancer. Alarming tomographic findings representing cancer in MS are the presence of a mass filling or replacing the gallbladder and local involvement of the liver with anterior surface focal protrusion [15].

## Conclusions

The understanding of MS and its different classifications/types are crucial for accurate diagnosis and optimal treatment. Gallstones are a common medical issue that may lead to chronic complications like MS; early diagnosis and appropriate management significantly impact the lives of patients. High suspicion of MS should be kept in the differential diagnosis in elderly patients who present with fatigue and obstructive jaundice, especially in the background of chronic cholecystitis. As in our case, the patient had a past medical history of recurrent cholecystitis and presented with fatigue, intermittent right upper quadrant pain and scleral icterus. Hence, physicians should keep MS in mind when managing patients with chronic gallstone cholecystitis. 
